# Awareness and utilization of smoking cessation clinics in Saudi Arabia, findings from the 2019 Global Adult Tobacco Survey

**DOI:** 10.1186/s13011-023-00543-0

**Published:** 2023-06-15

**Authors:** Sarah S. Monshi, Abdullah M. M. Alanazi, Ali M. Alzahrani, Abdulrhman A. Alzhrani, Turky J. Arbaein, Khulud K. Alharbi, Mansour Z. Alqahtani, Ali H. Alzahrani, Ahmed A. Elkhobby, Aljoharah M. Almazrou, Abdulmohsen H. Al-Zalabani

**Affiliations:** 1 Department of Health Services Management, College of Public Health and Health Informatics, Umm Al-Qura University, Makkah, Saudi Arabia; 2 Department of Respiratory Therapy, College of Applied Medical Sciences, King Saud Bin Abdulaziz University for Health Sciences, Riyadh, Saudi Arabia; 3 King Abdullah International Medical Research Center, Riyadh, Saudi Arabia; 4 Tobacco Control Program at Ministry of Health, Riyadh, Saudi Arabia; 5 Department of Family and Community Medicine, College of Medicine, Taibah University, Medina, Saudi Arabia

**Keywords:** Tobacco use, Smoking cessation, Smoking cessation services, Saudi Arabia

## Abstract

**Background:**

Tobacco use remains a leading cause of premature death. To combat tobacco use, the Ministry of Health (MOH) improved access to smoking cessation clinics (SCCs) by developing fixed SCCs and mobile SCCs, which move based on demand across locations. The goal of this study was to investigate awareness and utilization of SCCs among tobacco users in Saudi Arabia and the factors that influence their awareness and utilization.

**Method:**

This cross-sectional study used the 2019 Global Adult Tobacco Survey. Three outcome variables were employed, including tobacco users’ awareness of fixed SCCs, mobile SCCs, and utilization of fixed SCCs. Several independent variables were examined, including sociodemographic characteristics and tobacco use. Multivariable logistic regression analyses were performed.

**Results:**

One thousand six hundred sixty-seven tobacco users were included in this study. There were 60%, 26%, and 9% of tobacco users who were aware of fixed SCCs, aware of mobile SCCs, and visited fixed SCCs, respectively. The likelihood of being aware of SCCs increased among users residing in urban areas (fixed SCCs: OR = 1.88; 95% CI = 1.31–2.68; mobile SCCs: OR = 2.09; CI = 1.37–3.17) while it decreased among those reported self-employed (fixed SCCs: OR = 0.31; CI = 0.17–0.56; mobile SCCs: OR = 0.42; CI = 0.20–0.89). The likelihood of visiting fixed SCCs increased among educated tobacco users aged 25–34 (OR = 5.61; CI = 1.73–18.21) and 35–44 (OR = 4.22; CI = 1.07–16.64) while the odds of visiting SCCs decreased among those who were working in the private sector (OR = 0.26; CI = 0.09–0.73).

**Conclusion:**

The decision to quit smoking must be supported by an effective healthcare system that provides accessible and affordable smoking cessation services. Knowing the factors that influence the awareness and utilization of SCCs would help policymakers dedicate efforts targeting those who desire to quit smoking yet face limitations in using SCCs.

## Background

Tobacco use is a significant public health concern and a leading cause of premature death worldwide. Millions of people die annually due to the direct use of tobacco products and exposure to secondhand smoke [1]. Tobacco use is a significant risk factor for numerous chronic health disorders, including lung cancer, chronic obstructive pulmonary disease, cardiovascular diseases, and peripheral vascular disease [2]. The estimated global economic cost of tobacco use is $US 1.85 trillion [3]. In Saudi Arabia, the direct and indirect costs of smoking and secondhand smoke exposure were estimated at $17.20 billion in 2016 [4]. The recent Saudi national survey shows that the prevalence has continued to increase. As of 2019, the overall prevalence of tobacco use among those aged > 15 was 24% [5]. The patterns of tobacco use include conventional cigarettes, smokeless products, waterpipes (also called hookah or shisha), heated tobacco products (HTPs), and electronic cigarettes (e-cigarettes) [6].

In response to this public health problem, the Saudi government has made significant efforts to enact and implement tobacco control policies. In 2005, Saudi Arabia ratified the Framework Convention on Tobacco Control (FCTC) [7]. The FCTC consists of tobacco control policies targeting the demand and supply of tobacco to help nations overcome the issue of tobacco [7]. One of the policies that targeted the reduction of demand for tobacco is offering smoking cessation treatment and support, as stated in Article 14 of FCTC [8]. The WHO's recent report on the worldwide tobacco epidemic for 2019 highlighted that the Kingdom of Saudi Arabia is one of the leading countries in implementing the MPOWER strategies, particularly in helping individuals quit smoking through cessation services [9]. Of note, MPOWER strategy mainly targeting the demand for tobacco. It stands for (M) monitoring tobacco use and prevention policies, (P) protecting people from tobacco smoke, (O) Offering help to quit smoking, (W) warning about the danger of tobacco, (E) enforcing bans on tobacco ads, promotion, and sponsorship, and (R) raising tobacco price [10].

Quitting smoking can significantly reduce the risk of developing smoking-related diseases and improve overall health and quality of life [11]. On average, persons who stopped smoking before the age of 40 can avoid 90% of the tobacco-induced extra risk of mortality during their coming decades [12]. Although most smokers want to quit, they usually try to quit alone without medical support, leading to a low success rate [11]. Behavioral counseling and medications can increase the likelihood of success, highlighting the importance of making these services available to smokers [13]. Dedicated smoking cessation clinics (SCCs) can provide more comprehensive counseling and treatment.

In Saudi Arabia, these services are offered through the Ministry of Health (MOH). There are 500 SCCs with specific geographical locations distributed across Saudi Arabia [14]. MOH provides free-of-charge medical consultations, pharmaceutical medications, behavioral therapy, and follow-up care [15]. Additionally, to improve access to SCCs, the MOH developed 98 mobile SCCs that move based on demand across different locations and regions in Saudi Arabia [14, 15]. Moreover, MOH offers a support call center to provide periodic calls to check on progress, monitor, guide, and respond to inquiries from smokers and ex-smokers [9]. Since 2005, when the Saudi government ratified the FCTC, anti-smoking campaigns were run to raise awareness toward smoking cessation services [16]. Evidence shows that MOH SCCs have assisted 11,441 smokers to quit smoking in 2019 [16], resulting in smoking cessation rate of 31% [14].

The success of smoking cessation initiatives offered by the MOH is mainly dependent on the public's awareness of these programs and utilizing smoking cessation services. Approximately 42% of smokers in Saudi Arabia consider quitting smoking [5], but several barriers hinder them, including low self-efficacy, social pressure, and lack of knowledge about cessation services [8]. Although the growing number of SCCs in Saudi Arabia is promising [14], limited information is available about the awareness of SCCs among smokers, the utilization of clinics, and the effectiveness of these clinics. The goal of this study is to investigate awareness and utilization of SCCs among tobacco users in Saudi Arabia and the factors that influence their awareness and utilization.

## Method

### Data source

In this cross-sectional study, we utilized data from the 2019 Global Adult Tobacco Survey (GATS) collected by the Saudi MOH. The GATS gathered data related to tobacco use, tobacco control measures, and sociodemographic information. The GATS is a household survey that utilizes a multistage geographically clustered sampling design to collect nationally representative data [17]. A total of 12,800 households were sampled (household response rate = 98%). One adult, who was randomly chosen from the selected household, completed the questionnaire. Of the sampled households, 11,381 individuals completed the survey (person-level response rate = 96.2%) [5].

### Study participants

Our study focuses on adult tobacco users, defined as male and female participants aged 15 years and above who used any tobacco products daily or occasionally.

### Outcome variables

This study employed three outcome variables to measure tobacco users’ awareness and utilization of SCCs. The first outcome variable measured was tobacco users’ awareness of fixed SCCs, operationalized as users’ knowledge of the existence of fixed SCCs in their area. This variable was measured through the question, “Have you heard of fixed smoking cessation clinics in your area that provides assistance to quit smoking?” The second outcome variable measured was tobacco users’ utilization of fixed SCCs, operationalized as whether a smoker had visited fixed SCCs for assistance in quitting. This variable was measured through the question, "Have you visited a fixed smoking cessation clinic in your area to provide you with assistance to quit smoking?" Tobacco users who did not answer this question were assumed to have not visited an SCC. The third outcome variable measured was tobacco users’ awareness of mobile SCCs, operationalized as users’ knowledge of the existence of mobile SCCs in their area. This variable was measured through the question, “Have you heard of mobile smoking cessation clinics in your area that provides assistance to quit smoking?” The responses for the three questions were dichotomously recoded (0 & 1). It should be noted that the questions used to measure the outcome variables were not part of the standard GATS questionnaire; however, they were customized in purpose by the MOH in Saudi Arabia.

### Independent variables

The study considered several binary and categorical independent variables to examine their influence on the outcome variables. These variables included demographic characteristics such as age, gender, education, employment status, marital status, and place of residence. Additionally, the study included variables pertaining to the use of different forms of tobacco products, including cigarette, waterpipe, smokeless, and emerging tobacco products: heated tobacco products and electronic cigarettes. The inclusion of these variables was aimed at providing a comprehensive understanding of the relationship between the demographic and tobacco use characteristics and the outcome variables.

### Statistical analysis

We conducted univariate and bivariate analyses to evaluate the distribution of the study variables. Moreover, we created spatial maps to visualize the regional distribution of tobacco users who answered affirmatively to the outcome variables in the 13 administrative regions of Saudi Arabia. To determine the predictors of the outcome variables, we performed multivariable logistic regression analyses. These analyses consisted of two models for each outcome variable: Model I, which did not consider the impact of tobacco products, and Model II, which accounted for the influence of tobacco products. The association between the outcome variables and the dependent variables was estimated, controlling for all other variables. Complex sample design features were incorporated into all analyses to provide precise sample estimate [18]. All statistics and standard errors were weighted to account for the complex sample design used in the GYTS. The maps and analyses were conducted using STATA 17 [19]. This research has been granted IRB approval from Umm Al-Qura University (Approval No. HAPO-02-K-012-2022-11-1338).

## Results

From 2019 GATS, 1,667 participants were identified as tobacco users and were included in this study. Of the included sample, (36.11%) were from the age group (25–34 years), (91.3%) were males, (46.36%) had completed a high school or equivalent degree, (68.75%) were residing in urban areas, and (40.11%) were employees at the governmental sector. The majority of them were using cigarettes (89.02%), followed by waterpipes (30.11%). Overall, 60%, 26%, and 9% of the included sample were aware of fixed SCCs, aware of mobile SCCs, and visited fixed SCCs, respectively (Table 1).

**Table 1:** Demographic characteristics of tobacco users included in the study using 2019 GATS, Saudi Arabia (*N* = 1,667)

Characteristics	N	%
**Age groups**		
15–24	313	18.78
25–34	602	36.11
35–44	493	29.57
45 +	259	15.54
**Gender**		
Female	145	8.7
Male	1522	91.3
**Education**		
No formal education	140	8.43
Middle school and less	241	14.51
High school or equivalent degree	770	46.36
College or higher education	510	30.7
**Residence**		
Rural	521	31.25
Urban	1146	68.75
**Marital status**		
Not married	560	33.65
Married	1104	66.35
**Employment status**		
Government	663	40.11
Private	340	20.57
Self-employed	187	11.31
Student	175	10.59
Housewife	72	4.36
Unemployed	216	13.07
**Tobacco Products**		
Waterpipe		
Yes	502	30.11
No	1165	69.89
Cigarettes		
Yes	1484	89.02
No	183	10.98
Smokeless		
Yes	86	5.16
No	1581	94.84
HTP & E-cigarettes		
Yes	60	3.6
No	1607	96.4
**Outcomes of Interest**		
Aware of Smoking Cessation Clinics		
Yes	985	60
No	682	40
Visited Smoking Cessation Clinics		
Yes	148	9
No	1519	91
Aware of Mobile Smoking Cessation Clinics		
Yes	424	26
No	1243	74

*HTP & E-cigarettes* Heated Tobacco Products and Electronic Cigarettes

**Fig. 1:**
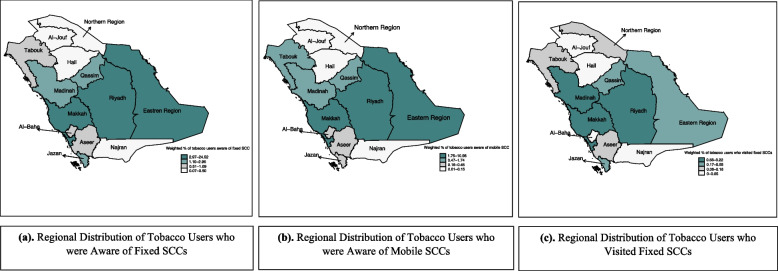
Regional distribution of tobacco users in relation to awareness and utilization of smoking cessation clinics using 2019 GATS, Saudi Arabia

Figure 1 represents the regional distribution of tobacco users based on the three outcomes of interest: awareness of fixed SCCs, mobile SCCs, and visiting fixed SCCs. Figure 1a showed that Makkah, Riyadh, and Eastern region had the highest percentages of tobacco users who were aware of fixed SCCs while Al-Jouf, Hail, Najran, and Northern region had the lowest percentages. Similarly, Makkah, Riyadh, and the Eastern region had the highest percentages of tobacco users who were aware of mobile SCCs. On the other hand, Al-Jouf, Hail, Najran, and the Northern region had the lowest percentages (as shown in Fig. 1b). In addition, a high percentage of tobacco users residing in Tabouk were more aware of mobile SCCs than fixed SCCs. Figure 1c shows the regional distribution of tobacco users who visited SCCs, with the highest percentages in Makkah, Madinah, and Riyadh, and the lowest in Al-Jouf, Hail, and Najran.

Among the tobacco users, (60%) were aware of fixed SCCs. The likelihood of being aware of fixed SCCs increased among tobacco users residing in urban areas (Odds Ratio [OR] = 1.88; 95% Confidence Interval [CI] = 1.31–2.68) and among those who were educated, in particular, those who completed middle school and less (OR = 4.24; CI = 2.14–8.42), high school or equivalent degree (OR = 5.03; CI = 2.56–9.88), or college or higher education (OR = 7.98; CI = 3.93- 16.19). However, the likelihood of being aware of fixed SCCs decreased among self-employed tobacco users (OR = 0.31; CI = 0.17–0.56) or those working in the private sector (OR = 0.38; CI = 0.23 – 0.61). With including the tobacco products in model II, the likelihood of being aware of fixed SCCs remained higher among educated tobacco users and those residing in urban areas and lower among those working in the private sector or were self-employed. In addition, the model showed that the likelihood of being aware of fixed SCCs decreased among unemployed tobacco users (OR = 0.5; CI = 0.27–0.94) and those reported using smokeless tobacco (OR = 0.47; CI = 0.27–0.82) (Table 2).

**Table 2:** Factors associated with awareness of smoking cessation clinics among tobacco users using 2019 GATS, Saudi Arabia (*N* = 1,667)

Variables	Model I		Model II	
	**OR**	**95% CI**	**OR**	**95% CI**
**Age Groups**				
15–24	Ref		Ref	
25–34	0.76	0.43–1.36	0.71	0.39–1.3
35–44	0.74	0.39–1.41	0.7	0.36–1.36
45 +	0.65	0.33–1.29	0.68	0.33–1.37
**Gender**				
Female	Ref		Ref	
Male	0.64	0.27–1.52	0.73	0.3–1.75
**Education**				
No formal education	Ref		Ref	
Middle school and less	4.24	2.14–8.42 **	4.68	2.31–9.49 **
High school or equivalent degree	5.03	2.56–9.88 **	5.11	2.55–10.24 **
College or higher education	7.98	3.93- 16.19 **	7.77	3.75–16.11 **
**Residence**				
Rural	Ref		Ref	
Urban	1.88	1.31–2.68 **	1.7	1.18–2.45 **
**Marital status**				
Not married	Ref		Ref	
Married	1.28	0.84–1.97	1.29	0.83–2.01
**Employment status**				
Government	Ref		Ref	
Private	0.38	0.23–0.61 **	0.38	0.23–0.62 **
Self-employed	0.31	0.17–0.56 **	0.31	0.17–0.56 **
Student	0.73	0.34–1.58	0.6	0.26–1.36
Housewife	0.33	0.1–1.07	0.36	0.12–1.24
Unemployed	0.55	0.3–1	0.5	0.27–0.94 *
**Tobacco Products**				
Waterpipe				
No	–	–	Ref	
Yes	–	–	1.18	0.76–1.82
Cigarettes				
No	–	–	Ref	
Yes	–	–	0.95	0.52–1.73
Smokeless				
No	–	–	Ref	
Yes	–	–	0.47	0.27–0.82 **
HTP & E-cigarettes				
No	–	–	Ref	
Yes	–	–	2.25	0.90–5.65

Association between the outcome and the independent variable was estimated, holding all other variables constant

*OR* Odds Ratio, *CI* Confidence Interval, *Ref* Reference Group, *HTP & E-cigarettes* Heated Tobacco Products and Electronic Cigarettes

^*^p < 0.05, ** *p* ≤ 0.01

There were (9%) of tobacco users who visited fixed SCCs. The likelihood of visiting fixed SCCs increased among tobacco users who were 25–34 years of age (OR = 5.61; CI = 1.73–18.21) or 35–44 years of age (OR = 4.22; CI = 1.07–16.64), among those completed middle school and less (OR = 16.02; CI = 2.85–89.89), high school or equivalent degree (OR = 15.22; CI = 2.69–86.14), or college or higher education (OR = 15.38; CI = 2.74–86.43), and among those whose employment status was a student (OR = 4.53; CI = 1.34–15.31). On the other hand, the odds of visiting fixed SCCs decreased among tobacco users who reported working in the private sector (OR = 0.26; CI = 0.09–0.73). Including the tobacco products in Model II did not show significant results; however, the findings for the tobacco users, who were 35–44 years of age, and those who were students, became nonsignificant (Table 3).

**Table 3:** Factors associated with tobacco users’ visits to smoking cessation clinics using 2019 GATS, Saudi Arabia (*N* = 1,667)

Variables	Model I		Model II	
	**OR**	**95% CI**	**OR**	**95% CI**
**Age Groups**				
15–24	Ref		Ref	
25–34	5.61	1.73–18.21 **	4.45	1.38–14.42 *
35–44	4.22	1.07–16.64 *	3.2	0.8–12.79
45 +	3.65	0.89–14.92	2.78	0.7–10.99
**Gender**				
Female	Ref		Ref	
Male	1.66	0.63–4.39	1.61	0.55–4.75
**Education**				
No formal education	Ref		Ref	
Middle school and less	16.02	2.85–89.89 **	16.73	2.98–93.93 **
High school or equivalent degree	15.22	2.69–86.14 **	15.01	2.59–87.03 **
College or higher education	15.38	2.74–86.43 **	14.8	2.6–84.35 **
**Residence**				
Rural	Ref		Ref	
Urban	1.08	0.54–2.19	1.21	0.62–2.34
**Marital status**				
Not married	Ref		Ref	
Married	0.92	0.41–2.09	0.96	0.42–2.2
**Employment status**				
Government	Ref		Ref	
Private	0.26	0.09–0.73 **	0.26	0.09–0.74 *
Self-employed	0.72	0.23–2.23	0.68	0.19–2.51
Student	4.53	1.34–15.31 *	3.42	0.99–11.83
Housewife	0.63	0.08–4.74	0.81	0.1–6.28
Unemployed	0.9	0.32–2.55	0.81	0.28–2.4
**Tobacco Products**				
Waterpipe				
No	–	–	Ref	
Yes	–	–	0.51	0.26–1.01
Cigarettes				
No	–	–	Ref	
Yes	–	–	2.24	0.37–13.65
Smokeless				
No	–	–	Ref	
Yes	–	–	1.37	0.57–3.29
HTP & E-cigarettes				
No	–	–	Ref	
Yes	–	–	2.86	0.94–8.69

Association between the outcome and the independent variable was estimated, holding all other variables constant

*OR* Odds Ratio, *CI* Confidence Interval, *Ref* Reference Group, *HTP & E-cigarettes* Heated Tobacco Products and Electronic Cigarettes

^*^p < 0.05, ** *p* ≤ 0.01

Tobacco users who were aware of mobile SCCs accounted for (26%). The likelihood of being aware of mobile SCCs increased among tobacco users who completed high school or equivalent degree (OR = 3.58; CI = 1.44–8.9), or college or higher education (OR = 3.24; CI = 1.27–8.26), and among those residing in urban areas (OR = 2.09; CI = 1.37–3.17). However, the likelihood of awareness decreased among self-employed tobacco users (OR = 0.42; CI = 0.2–0.89). No significant change in findings was observed when adding tobacco products to model II (Table 4).

**Table 4:** Factors associated with awareness of mobile smoking cessation clinics among tobacco users using 2019 GATS, Saudi Arabia (*N* = 1,667)

Variables	Model I		Model II	
	**OR**	**95% CI**	**OR**	**95% CI**
**Age Groups**				
15–24	Ref		Ref	
25–34	1.44	0.71–2.92	1.4	0.67–2.91
35–44	1.08	0.52–2.23	1.01	0.47–2.15
45 +	2.19	0.1–4.8	2.17	0.96–4.90
**Gender**				
Female	Ref		Ref	
Male	0.62	0.27–1.41	0.76	0.32–1.78
**Education**				
No formal education	Ref		Ref	
Middle school and less	2.06	0.80–5.31	2.03	0.77–5.36
High school or equivalent degree	3.58	1.44–8.90 **	3.49	1.42–8.60 **
College or higher education	3.24	1.27–8.26 *	2.90	1.13–7.38 *
**Residence**				
Rural	Ref		Ref	
Urban	2.09	1.37–3.17 **	2.17	1.43–3.29 **
**Marital status**				
Not married	Ref		Ref	
Married	0.89	0.57–1.41	0.81	0.51–1.29
**Employment status**				
Government	Ref		Ref	
Private	0.80	0.49–1.30	0.78	0.48–1.28
Self-employed	0.42	0.20–0.89 *	0.38	0.18–0.81 *
Student	0.84	0.36–1.92	0.58	0.24–1.40
Housewife	0.28	0.07–1.20	0.33	0.08–1.47
Unemployed	0.55	0.28–1.06	0.51	0.27–1.00
**Tobacco Products**				
Waterpipe				
No	–	–	Ref	
Yes	–	–	1.20	0.76–1.89
Cigarettes				
No	–	–	Ref	
Yes	–	–	1.07	0.56–2.03
Smokeless				
No	–	–	Ref	
Yes	–	–	0.93	0.43–2.01
HTP & E-cigarettes				
No	–	–	Ref	
Yes	–	–	2.24	0.92–5.41

Association between the outcome and the independent variable was estimated, holding all other variables constant

*OR* Odds Ratio, *CI* Confidence Interval, *Ref* Reference Group, *HTP & E-cigarettes* Heated Tobacco Products and Electronic Cigarettes

^*^p < 0.05, ** *p* ≤ 0.01

## Discussion

This study explored the awareness of SCCs and utilization of SCCs among tobacco users from national GATS 2019 data in Saudi Arabia. In general, greater awareness of fixed and mobile SCCs and utilization of SCCs were among those with higher educational attainments and residing in urban areas of Makkah, Riyadh, and Eastern regions. On the other hand, less awareness was found among private employment, self-employed, and unemployed tobacco users. In addition, a lower prevalence of fixed and mobile SCCs awareness was found in the northern and southern regions of Saudi Arabia. Of note, there were no differences among tobacco product uses with respect to the awareness of fixed and mobile SCCs and utilization of SCCs, except for smokeless tobacco users who were less aware of fixed SCCs.

Our findings on the awareness and utilization of SCCs were not different from other countries. For example, the awareness of smoking cessation services among Indians was higher among educated people and those residing in urban areas [20]. In the United States, the awareness and utilization of national Quitline were lower among less educated people and older adults [21]. Indeed, regional and district differences in awareness were also present in both countries [20, 21]. Although it is expected to have differences in the awareness and utilization of SCCs, especially in populated countries, such inequalities in SCCs awareness and utilization may be justified with significant outreach initiatives across the nation. Future smoking cessation initiatives should target northern and southern regions in Saudi Arabia to address the disparities in smoking cessation assistance.

Part of Saudi Arabia's agenda is to lower the national prevalence of tobacco use by offering national coverage of SCCs and mobile SCCs to all country residents [16]. In addition, mass media and community awareness about the harm of smoking and smoking cessation services are diffused, especially in healthcare, governmental, and educational settings [16]. However, several questions remain about which tobacco users are aware of and utilizing such smoking cessation services. According to these national findings, significant differences in awareness and utilization of SCCs were observed based on sociodemographic and geographical regions. For instance, the odds of the awareness and utilization of SCCs decreased among those who reported working in the private sector or self-employed. Social norms within workplaces, such as insufficient enforcement of smoke-free policies or stress related to high job demand, could explain this finding [22]. In addition, this study found limited awareness of SCCs among smokeless tobacco users, which could be interpreted by the misperception about the harmful effect of smokeless tobacco products [23].

The study findings encourage the development of tailored smoking cessation services for less aware residents who use tobacco and enhance the implementation of ongoing surveillance, based on specific measurable outcomes, to track awareness and utilization of SCCs to evaluate such initiatives [24]. Engaging private employers, using digital solutions, and designing culturally tailored campaigns that are evidence-based interventions in smoking cessation services shall help tobacco users with different sociodemographic backgrounds and regional areas to accept, access, and utilize SCCs [25–27]. It is also important to recognize that greater awareness and utilization of SCCs do not imply that such services were effective in helping tobacco users quit. Tobacco use is a chronic relapsing disorder that requires a significant emphasis on therapeutic and behavioral interventions besides follow-up programs [28]. National initiatives may expand to assess smoking cessation services from providers’ and tobacco users’ perspectives to understand the quality of services and fill its gaps.

## Limitations

This study has several limitations. First, the study used GATS, which relied on a self-reporting questionnaire. This type of instrument may be subject to recall and subject desirability bias, which may affect the study findings. Second, the lack of surveillance of tobacco control policies and programs at the local-level limited our ability to control the confounding effect of interventions that might have been implemented before or during data collection. For instance, there might be an anti-tobacco campaign that was conducted at a particular time and region, which may have influenced the participants' responses. Finally, the questions used to measure outcomes were not a part of the standard GATS; they were designed by the Saudi MOH. Although these questionnaire questions are essential to assess the healthcare system's readiness to support those willing to quit smoking, the validity of these questions needs to be examined. Future research could contribute to examining the validity of these questionnaire questions to expand research in this field.

## Conclusion

Smoking cessation remains one of the effective tobacco control interventions that reduces risks of chronic diseases and alleviates the economic burden associated with tobacco use. However, quitting smoking is not an easy decision when changing unhealthy behavior. It requires a supportive healthcare system that provides accessible and affordable smoking cessation services to those who desire to quit smoking. Knowing the factors that influence the awareness and utilization of SCCs in Saudi Arabia would help policymakers dedicate efforts and allocate budgets targeting those with limitations in utilizing SCCs and design tailored interventions to enhance smoking cessation.

## Data Availability

The dataset “2019 Global Adult Tobacco Survey” used in this study was obtained from the Tobacco Control Program at the Ministry of Health, Saudi Arabia. It could be accessed upon request, which should be sent to the Ministry of Health.

## Abbreviations:

CI: Confidence Interval
e-cigarettes: Electronic Cigarettes
FCTC: Framework Convention on Tobacco Control
GATS: Global Adult Tobacco Survey
HTPs: Heated Tobacco Products
MOH: Ministry of Health
OR: Odds Ratio
SCCs: Smoking Cessation Clinics
